# Management of haemorrhoids through siddha formulation *Kuppaimeni Samoola*

**DOI:** 10.6026/9732063002001461

**Published:** 2024-11-05

**Authors:** Gayatri R, Vinayak S, Gnanaraj Johnson Christian, Hrudayakumari P, Ramamurthy Murugan, Elansekaran Selladurai, Srinivasan V, Shakthi Paargavi A, Saravanasingh Karan Chand Mohan Singh, Karthi Senthil

**Affiliations:** 1 Department of Noi Naadal, National Institute of Siddha, Tambaram, Sanatorium, Chennai - 47, India; 2 Siddha Central Research Institute (SCRI), Chennai, Tamil Nadu, India; 3 Department of Udal Koorugal, National Institute of Siddha Chennai, Tamil Nadu, India

**Keywords:** *Kuppaimeni samoola chooranam*, hemorrhoids, *moolam*, *siddha*

## Abstract

Hemorrhoids are a very common anorectal condition characterized by symptomatic enlargement and distal dislodgment of anal cushions which mainly affects the quality of life. Siddha herbal medicine Kuppaimeni (*Acalypha Indica*) is indicated for haemorrhoids, was used in this pilot study to catalogue the feasibility of Kuppaimeni samoola choornam in the management of first, second, and third- degree internal Hemorrhoids, along with a dietary regimen, of including two fig fruits in the daily diet. Ten participants were selected for the study and they underwent medication with a drug administration calendar period of forty - eight days. Prognosis was assessed by using Hemorrhoids Symptoms Severity (HSS) Scoring. The study findings of KSC are: among the ten cases, eight subjects got complete relief from symptoms after 48 days of medication. HSS scoring reduced to zero for all (8) first and second degree hemorrhoid cases and these 8 cases never had repeated episodes in follow-up time (5 years of follow-up after withdrawing medication). This study suggests the feasibility of using KSC in treating first and second-degree internal hemorrhoids.

## Background:

Hemorrhoids are a disease condition defined well in medical history and has disturbed humanity's quality of life since ancient times. From time immemorial convincing descriptions of Hemorrhoids are available, in Mesopotamian civilization dating as well as in the Holy Bible in which the term 'emerods' is mentioned [arch. Hemorrhoids] and Indian Medical texts defined under "c" strengthen their position as a condition about ano - rectal region of the ages. Ancient treatment for Hemorrhoids varied between intake of herbal concoctions, hot iron cauterization, leech application and "transfixing them with a needle and tying them with a very thick and large woolen seton" [1, 2]. As per ICD 11 DB, 60 haemorrhoids are defined as a prolapse with symptomatic enlargement and distal dislodgment of anal cushions associated with bleeding and painful swelling in the anal canal. Internal haemorrhoids are swollen veins inside the anal canal originating above the pectinate line and covered by mucous membranes [3]. It can be considered as a global burden as it leads to both bodily and psychological suffering for patients around the world. Many factors lead to this condition among which constipation and prolonged straining are very common. The abnormal dilatation and distortion of the vascular channel, together with destructive changes in the supporting connective tissue within the anal cushion, is a chief finding of haemorrhoidal disease. Inflammations, as well as hyperplasia, are evident in haemorrhoids [4]. Vascular tissue present in the anal sub mucosa (haemorrhoids) is generally made of loose connective tissue, which will help in making stool bulk continence enough to pass. They typically occur at three locations: left lateral, right ventral, and right dorsal sites, receiving blood from superior, middle, and inferior haemorrhoidal arteries. Venous drainage is through the inferior and middle haemorrhoidal veins, which ultimately drain into the iliac veins. Internal Hemorrhoids disease is classified into four based on the degree of prolapse: grade I Hemorrhoids show a protrusion without prolapse; grade II Hemorrhoids prolapse during defecation and ascend naturally without manual reduction; grade III Hemorrhoids prolapse and require manual reduction; grade IV Hemorrhoids prolapse and are not reducible [5].

First- degree Hemorrhoids are often considered manageable with diet and lifestyle modifications. Evidence states fibre rich diet reduces hemorrhoid symptoms, complaints of bleeding on strain reduced in terms of 50% relative risk (RR) [6]. There is number of medicines available in the market for haemorrhoidal symptoms, but these topical agents typically contain low-dose anaesthetics, steroids, protectants, antiseptics, and astringents [7]. Further, the utility of conservative measures is rarely scientifically supported by adequate trials [8]. Additional therapeutic options that can provide prompt relief for first-degree hemorrhoids include rubber band ligation and injectable sclerotherapy. However, only those who do not respond to non- surgical procedures should undertake these more intrusive therapies. Rubber band ligation (RBL) is the preferred method for treating second-degree hemorrhoids and is the most commonly used non-surgical procedure. According to a recent study conducted by Robin et al in the Netherlands, 90% of surgeons stated that they begin treatment for low-grade haemorrhoidal illness by using RBL as the initial approach [9]. Different treatment modalities are available, like expectant medical therapy, rubber band ligation, manual dilatation, cryosurgery, infrared coagulation, and operative treatments like formal hemorrhoidectomy [10]. However, there is a high risk of recurrence or developing an infection of the wound after the surgical correction. Risks of Milligan-Morgan hemorrhoidectomy are pain - which differs from no symptom at all to severe; retention of urine - 7% of patients required catheterization secondary hemorrhage - 1.2% of patients, and development of abscess or fistula [11]. Also, a long time hospitalization is needed. Even though there are numerous treatment procedures, the balance continues to be sought between lasting effect, minimization of pain, and preservation of anorectal function. So, there is a need to develop an alternate treatment strategy for this condition because this condition results in physical and psychological distress of the patient.

Siddha system of medicine is one of the traditional Indian medical systems, and it has many indicated drugs for the condition of Hemorrhoids which are in practice. Plain Kuppaimeni plant crude herbal powder is a natural remedy to treat Hemorrhoids (*Acalypha Indica*) [13]. The plant *A. indica* belongs to the *Euphorbiaceae* family; it grows in India, Indian Ocean islands, Southeast Asia, Oceania, East Africa, and southern Africa, including South Africa and is introduced into warmer parts of the world [14]. Likewise, computational studies also prove that lead molecules present in *Self-Assessment Questionnaire* are having anti - inflammatory activity [15]. Since *A. indica* is found to be anti - inflammatory as mentioned before and chronic serious inflammatory reaction involving both the vascular part (sinusoidal wall) and non-vascular part (supportive tissue) has been demonstrated in Hemorrhoids [16]. The inflammatory process renders the arterioles of the lamina propria of the nodule vulnerable to erosion during defecation with resultant hemorrhage [17]. Hence, an herb like *A. indica*, with good anti-inflammatory activity and anecdotal evidence can be considered a candidate drug of choice for the management of conditions like haemorrhoidal disease. *Ega Mooligai Prayogam* (Mono Herbal Therapy) in Siddha system is indicated for the treatment of several conditions so, if a single herb and a simple diet significantly improve this condition, it will be helpful for the individuals suffering from this condition. Therefore, it is of interest to report the efficacy of *Kuppaimeni samoola choornam* [13] in the management of internal Hemorrhoids (*Moolam*).

## Methods:

**Figure 1:**
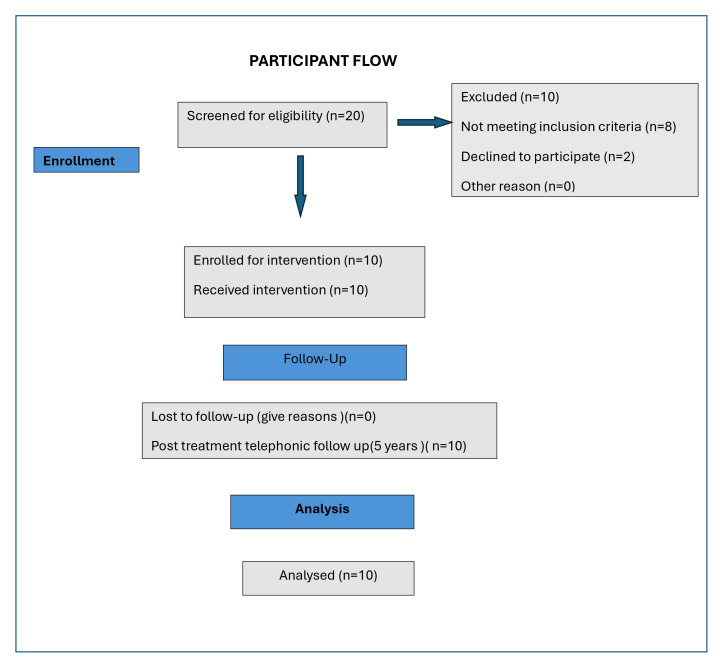
Participant flowchart

This study was an interventional study (pilot clinical trial) done after getting approval from the Institutional Ethics Committee of the National Institute of Siddha and the same was registered in the Clinical Trial Registry of India. Figure 1 The study was conducted in patients of the age group 20-50 years who visited OPD and IPD of Ayothidoss Pandithar Hospital, National Institute of Siddha, Chennai, between 2017 and 2018, reported complaints of internal Hemorrhoids up to third degree on examination. Patients with complaints of rectal varices, anal fissures and fistula - in - ano, malignancies, fourth-degree Hemorrhoids and other terminally ill patients were to be excluded from the study. Selected subjects were subjected to proctoscopic examination through which diagnosis confirmation and grade of Hemorrhoids were ascertained. After screening 20 subjects consecutively, 10 subjects who were suitable as per eligibility criteria were enrolled for study. The trial drug was *Kuppaimeni Samoola chooranam* (powder prepared by *Acalypha Indica* (*kuppai meni*) whole plant shade dried and powdered form). Medicine was procured from GMP certified pharmacy. The dose of the drug was 2 grams twice daily, along with water for 48 days. Patients were also advised to include 2 Fig fruits in their daily diet. Treatment adherence was documented through a drug compliance form. Clinical assessment was done during each visit from baseline every week; for outcome assessment, the scoring of Hemorrhoids Symptoms Severity (HSS) by Karolinska University and proctoscopic examination was used at baseline, interim and endpoint [18]. The proctoscopic examination was used at baseline, interim and endpoint. *Self-Assessment Questionnaire* [19] was used to assess post treatment follow up after 5 years. The questions are:- At the moment, do you feel your symptoms from your hemorrhoids are:

[1] Cured or improved compared with before treatment; or

[2] Unchanged or worse compared with before treatment?"

## Results:

Twenty participants were screened for eligibility based on the selection criteria. Consecutive sampling was employed for selecting participants. After going through the selection criteria, eight participants were excluded since they did not satisfy the inclusion criteria and two subjects declined to participate and only ten participants were enrolled as trial participants. Participants diagnosed with internal Hemorrhoids with features like pain in the anal region, itching, bleeding per rectum, soiling and protrusion of pile mass through the anus were subjected to trial medicine.

Two participants had third-degree Hemorrhoids, and four had second-degree and first- degree. Primary and secondary outcome were analyzed. Pain and bleeding per rectum while defecating were the most common complaints presented by all patients; other presenting features were itching in anal region and manual reduction of pile mass and soiling were the variables in the HSS assessment scoring. Recruitment and follow-up were done during 2017 and 2018; after that, a drugless follow-up over telephonic conversation was also done after five years in 2023 to document the trial drug's long-term effect and to note recurrence, if any. It is tabulated in Table 4. While analyzing the outcomes, it was found that pain and bleeding were the main presenting complaints of participants. Eight among ten subjects had complaints of bleeding and painful defecation daily, and for all of them, these features were markedly reduced after 24 days and completely relieved after 48 days of medication. Among ten cases, 4 of them had itching in the anal region that got completely relieved after 24 days of medication. Two subjects who had manual polyp reduction while defecation did not show much improvement; for one subject, frequency got reduced, but for the other, there was no improvement in manual polyp reduction. Assessment on the 24th day and 48th day of medication through HSS scoring is shown in Table 1, Table 2 and Table 3. Drugless follow-up showed that the medicine is effective for first- degree and second-degree Hemorrhoids with a status of no recurrence of hemorrhoidal disease. However, one participant with third-degree ended up in surgical management, and the other still had complaints but in a manageable status. None of the participants showed any adverse event.

## Discussion:

Regardless of low prevalence and morbidity, hemorrhoids disease has an influences quality of life and can be managed with a multitude of invasive and non - invasive procedures based on the clinical features. One general guiding principle is that the least-invasive approaches should be considered first. Specific choices of treatments depend on patients' age, severity of symptoms, and comorbidities [16].

## Principal findings:

A range of invasive procedures, namely rubber band ligation, sclerotherapy, infrared coagulation, stapled hemorrhoidoplexy, Doppler-guided hemorrhoid artery ligation, *etc.,* are available modalities of treatment. Non-invasive procedures are always cost-effective and show more patient compliance than invasive procedures [17]. In this study, we have treated ten hemorrhoids (n=4 grade I, n=4 grade II and n=2 grade III) patients with *Kuppaimeni samoola churanam* [13] and a dietary regimen of two fig fruits daily. All the first and second-degree cases responded with no recurrence even after an approximate drug-less follow-up of 5 years as mentioned in Table 4. This study reveals the trial drug is having an effect in reducing bleeding and pain while defecating. In hemorrhoids, there will be inflammation with the erosion of cushions epithelium, which results in bleeding [20]. Hence, drugs with anti-inflammatory properties may be indicative. Likewise, the antioxidants protect and repair damaged anorectal tissues from free radicals. At the same time, the growth factors used in these combinations will promote cell renewal and thus ensue in the repair of affected tissues. Studies indicate that *A. indica* root extract is rich in various phytochemical constituents without adverse effect descriptors, which have anti- inflammatory properties.

## Possible mode of action:

Evidence suggests that *A. indica* is a potential anti-microbial, anti-diabetes, anti-inflammatory agent, larvicidal, antioxidant, and herb with wound healing properties. Analgesic property of *A. indica* was tested in rodent models which exhibited good results. A 21.51% and 30.64 % edema reduction was shown by 125 and 250 mg/kg of methanolic extract of *A. indica* in carrageenan-induced paw edema. Godipurge *et al.* (2015) reported that 400 mg/kg of polyphenolic-rich extract exhibited potential inhibition of paw volume (92.3%) than standard diclofenac sodium (61.5 %) of 0.9 mg/kg by the action on prostaglandin E2 production [11]. The major phytomolecules present in this herb are ramipril glucuronide, antimycin A, swietenine, quinone, oxprenolol, choline, bumetanide and fenofibrate. *Acalypha Indica* root methanolic extract (AIRME) showed potent free radical scavenging activity (DPPH and hydroxyl) and inhibition of lipid peroxidation ability. Improved antioxidant status with AIRME was further confirmed by its tissue-protective effects against acute inflammatory damage. Thus, AIRME may be considered as a drug of choice for conditions with inflammation [21].

## Implications for clinical practice:

KSC is not only mentioned in the Siddha system of medicine but also in the ethnomedical practices of Mosambique for the same indication of Hemorrhoids by crushing the plant and making decoction out of it [22].

This study also suggests KSC has the potential to reduce symptoms of Hemorrhoids, especially bleeding per rectum and painful defecation, which affect the quality of life of an individual. KSC effectively reduced the symptoms even after a few days of medication. The most highlighting factor is, among the ten cases, 8 subjects were completely relieved from symptoms after 48 days of medication, and these 8 cases never had repeated episodes in follow-up time (5 years of follow-up after withdrawing medication). No adverse events were reported for any of the cases.

## Conclusion:

From this feasibility study, it can be stated that this single herb is effective enough in alleviating the complaints of first-and second-degree Hemorrhoids. However, to generalize the effect, further trials should be done.

## Registration number and name of trial registry:

Obtained Institutional Ethics Committee Clearance from IEC- National Institute of Siddha (NIS/IEC/2016/11-27/14.10.2016) and the same was registered in the Clinical Trial Registry of India (CTRI/2017/03/008176).

## Sources of funding and other support:

This was not a funded study, no external funding organization was involved in the design and conduct of the study, the collection, management, analysis, and interpretation of the data, the preparation, review, or approval of the manuscript, or the decision to submit the manuscript for publication. Consent to participate and publish: Written informed consent was obtained from the participants. Declaration of competing interest: The authors declare no conflict of interest regarding the submitted work. The study was conducted at the National Institute of Siddha, Chennai, and Tamil nadu.

## Figures and Tables

**Table 1:** Hemorrhoids symptoms severity scoring - before treatment

**S.NO**	**Pain**	**Itching**	**Bleeding**	**Soiling**	**Frequency of manual polyp reduction**	**Total score**
1	3	0	3	0	0	6
2	2	1	1	0	3	7
3	3	0	1	0	3	7
4	3	0	3	0	0	6
5	3	0	3	0	0	6
6	3	1	3	0	0	7
7	3	1	3	0	0	7
8	3	1	3	0	0	7
9	3	0	3	0	0	6
10	2	0	3	0	0	5
(NEVER=0, <1/WEEK=1,1-6 TIMES A WEEK=2, EVERYDAY=3)

**Table 2:** Hemorrhoids grading and symptoms severity scoring - interim assessment after 24 days

**S.NO**	**Pain**	**Itching**	**Bleeding**	**Soiling**	**Frequency of manual polyp reduction**	**Total score**
1	2	0	2	0	0	4
2	2	0	1	0	3	6
3	0	0	1	0	3	4
4	2	0	1	0	0	3
5	3	0	2	0	0	5
6	2	0	2	0	0	4
7	2	0	2	0	0	4
8	3	0	1	0	0	4
9	3	0	1	0	0	4
10	1	0	0	0	0	1
(NEVER=0, <1/WEEK=1,1-6 TIMES A WEEK=2, EVERYDAY=3)

**Table 3:** Hemorrhoids symptoms severity scoring - after treatment

**S.NO**	**Pain**	**Itching**	**Bleeding**	**Soiling**	**Frequency of manual polyp reduction**	**Total score**
1	0	0	0	0	0	0
2	0	0	0	0	3	3
3	0	0	0	0	2	2
4	0	0	0	0	0	0
5	0	0	0	0	0	0
6	0	0	0	0	0	0
7	0	0	0	0	0	0
8	0	0	0	0	0	0
9	0	0	0	0	0	0
10	0	0	0	0	0	0
(NEVER=0, <1/WEEK=1, 1-6 TIMES A WEEK=2, EVERYDAY=3)

**Table 4:** Post treatment follow up as per post follow - up *Self-Assessment Questionnaire*

**S.No.**	**Hemorrhoids Grade**	**Post treatment follow up status**
1	Grade I	cured
2	Grade III	Unchanged, underwent surgery
3	Grade III	Unchanged
4	Grade I	cured
5	Grade I	cured
6	Grade II	cured
7	Grade II	cured
8	Grade I	cured
9	Grade II	cured
10	Grade II	cured
